# Supplementary material for: Splice-Junction-Based Mapping of Alternative Isoforms in the Human Proteome
Source: Cell Rep. Author manuscript; Available in PMC 2020 Jan 15. (PMC6961840; doi:10.1016/j.celrep.2019.11.026)
Supplement: 2 [file NIHMS1546469-supplement-2.zip › DF1/PXD010154/Lung/Lung_27_NA_AQLVIYAGPNTNSRK.pdf]

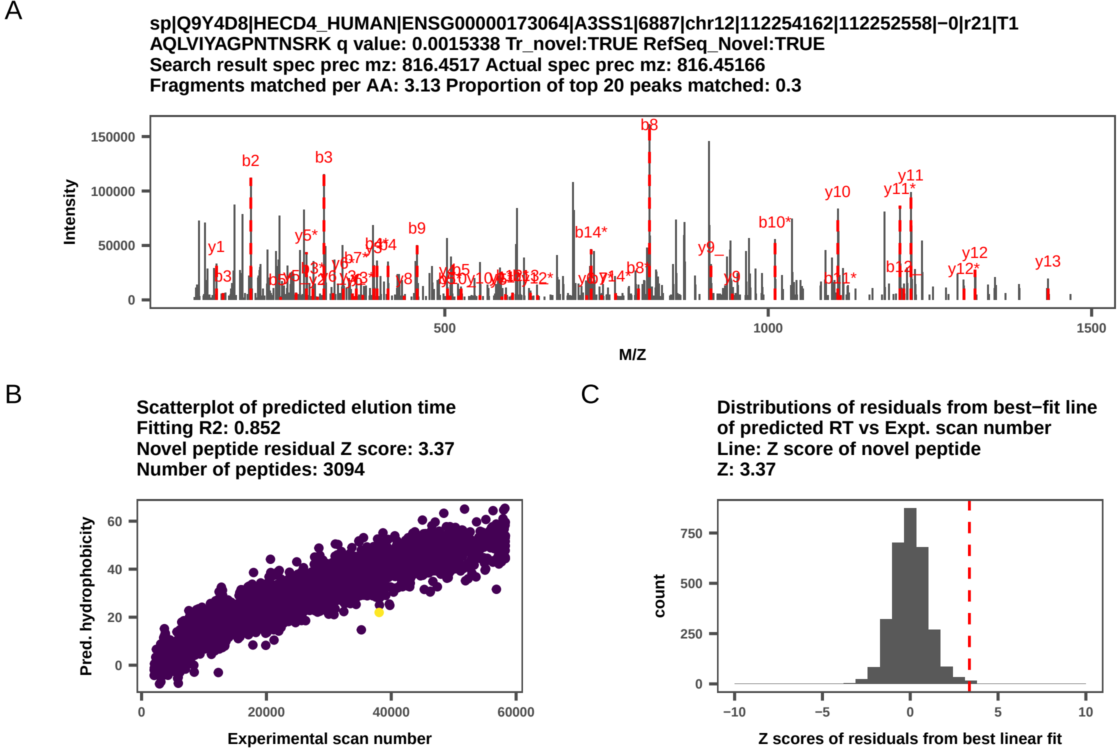
A
sp|Q9Y4D8|HECD4_HUMAN|ENSG00000173064|A3SS1|6887|chr12|112254162|112252558|−0|r21|T1
AQLVIYAGPNTNSRK q value: 0.0015338 Tr_novel:TRUE RefSeq_Novel:TRUE
Search result spec prec mz: 816.4517 Actual spec prec mz: 816.45166
Fragments matched per AA: 3.13 Proportion of top 20 peaks matched: 0.3
Intensity
150000
100000
50000
0
M/Z
500
1000
1500
b2
b3
b8
b9
y1
y5*
b10*
b14*
y10
y11
y11*
y12
y12*
y13
B
Scatterplot of predicted elution time
Fitting R2: 0.852
Novel peptide residual Z score: 3.37
Number of peptides: 3094
Pred. hydrophobicity
60
40
20
0
Experimental scan number
0
20000
40000
60000
C
Distributions of residuals from best−fit line
of predicted RT vs Expt. scan number
Line: Z score of novel peptide
Z: 3.37
count
750
500
250
0
Z scores of residuals from best linear fit
−10
−5
0
5
10
